# Automated Symbolic Orienting: The Missing Link

**DOI:** 10.3389/fpsyg.2012.00560

**Published:** 2012-12-17

**Authors:** Jelena Ristic, Mathieu Landry, Alan Kingstone

**Affiliations:** ^1^Department of Psychology, McGill UniversityMontreal, QC, Canada; ^2^University of British ColumbiaVancouver, BC, Canada

**Keywords:** attention, automaticity, reaction time, additive factors method, performance, behaviorally relevant stimuli

## Abstract

Attention can be controlled either exogenously, driven by the stimulus features, or endogenously, driven by the internal expectancies about events in the environment. Extending this prevailing framework, we (Ristic and Kingstone, 2012) recently demonstrated that performance could also be independently controlled by overlearned behaviorally relevant stimuli, like arrows, producing automated effects. Using a difficult target discrimination task within a double cuing paradigm, here we tested whether automated orienting engages selective attention, and if in doing so it draws on its own pool of attentional resources. Our data unequivocally support both possibilities, and indicate that human attention networks are uniquely specialized for processing behaviorally relevant information.

## Introduction

Control of human attention is routinely attributed to processes that occur exogenously (reflexively; Posner, 1980) or endogenously (volitionally; Jonides, 1981). While this framework has underscored a broad range of attention research in terms of the population studied (e.g., animals, infants, the aged, patients) and the techniques used (e.g., behavioral, TMS, ERP, fMRI; e.g., Brodeur et al., 1997; Bartolomeo and Chokron, 2002; Corbetta and Shulman, 2002; Dorris et al., 2002), it has struggled to explain recent data derived from the *model cuing task* (Posner, 1980), which has been foundational for experimentally invoking and measuring exogenous and endogenous orienting. Specifically, when behaviorally relevant symbolic stimuli, such as spatially nonpredictive arrows are used as attentional cues, the resultant data cannot be explained fully as engaging either exogenous or endogenous orienting (e.g., Gibson and Kingstone, 2006; Ristic and Kingstone, 2006). Like exogenous orienting, (Posner, 1980) attentional effects of nonpredictive arrows are found to emerge quickly in response to a spatially nonpredictive cue (e.g., Hommel et al., 2001; Ristic et al., 2002; Tipples, 2002); like endogenous orienting, (e.g., Muller and Rabbitt, 1989) attentional effects of arrow cues persist for up to 1 s without producing an inhibition of return effect (IOR; Posner and Cohen, 1984; McKee et al., 2007).

Ristic and Kingstone (2012) recently proposed that this gap between the prevailing theory that divides attentional processes to those that are exogenous and those that are endogenous and the data that cannot be explained by either process alone arises because spatially nonpredictive cues engage a novel and independent control mechanism called *automated symbolic orienting*. This new mechanism reflects a form of control that is derived from overlearning a cue’s meaning (e.g., typically a left- or right-pointing arrow reliably communicates left and right information), and thus provides a theoretical framework that can accommodate attentional effects of behaviorally relevant cues, like arrows. The aim of the current study is to test whether automated spatial orienting engages selective attention that enhances target’s perceptual discrimination (e.g., Hawkins et al., 1990), and if so, whether it draws on the same or different pool of attentional resources as exogenous and endogenous orienting (Klein, 2009).

Ristic and Kingstone (2012) ran two target detection experiments pairing a spatially nonpredictive central arrow with either an exogenous or an endogenous cue. Each cue had its standard effect on performance: exogenous peripheral cues produced early response time (RT) facilitation and then IOR (e.g., Posner and Cohen, 1984); central spatially predictive cues slowly gave rise to facilitation (e.g., Muller and Rabbitt, 1989); and nonpredictive arrow cues produced rapid and sustained facilitation (e.g., Hommel et al., 2001; Tipples, 2002).

The critical discovery was that the exogenous and endogenous effects operated independent of, and combined additively with, the effects of nonpredictive arrow cues. Ristic and Kingstone proposed the thesis that the arrows invoke a unique form of automated symbolic orienting that arises from long-term reliable contingencies between arrows and their spatial meaning. This conceptualization provides a way to reconcile, both empirically and theoretically the discordant interpretations that have been put forward this past decade with regard to the measured effects of nonpredictive directional cues (see for example Frischen et al., 2007).

Admittedly, the proposal of a new control system is a strong argument and as such warrants close scrutiny. Perhaps the most fundamental question one can pose about the Ristic and Kingstone study is whether their use of a simple target detection task provided a measurement of spatial attention at all. Detection tasks are vulnerable to the influences of two key factors that are not rate limited by attentional processing. First, detection of a salient target does not *require* the commitment of attentional resources, or at the very least, the selective commitment of spatially focused attention; processes that are needed when one is required to perceptually discriminate target’s features, indexing the key functional consequence of attentional selection – the boosting of the target’s early sensory processing (Luck et al., 1994; Hopfinger and Mangun, 1998). Second, RT effects from detection tasks are susceptible to contamination by decision factors (e.g., Shaw, 1978, 1984). Namely, it is possible that the RT advantage for cued targets, which is interpreted as the key indicator of attentional orienting, might instead reflect a bias to respond to the cued location. Determining whether Ristic and Kingstone’s data reflected attentional engagement thus carries important theoretical commitments, for their proposal of a novel form of orienting both questions past data that were situated within the traditional framework of exogenous and endogenous orienting, and presents exciting new avenues for future research.

These issues can be addressed by comparing participants’ performance using a simple target detection task *and* a difficult target discrimination task, as target discrimination provides a way to assess the engagement of selective attention and thus index the perceptual processes associated with target discrimination (e.g., Hopf et al., 2002). In the present study, we presented participants with a simultaneous double cue task, pairing a central spatially nonpredictive arrow with either an exogenous spatially nonpredictive peripheral (NP) onset cue (NP group) or an endogenous spatially predictive symbolic cue (Predictive Central, PC group). Crucially, in addition to a target detection task, participants also performed a difficult target discrimination task (cf. Berger et al., 2005, Experiment 4). This allowed us to test whether automated orienting engages selective attention by assessing its impact on a target’s perceptual discrimination. Finally, as target discrimination invokes a greater demand for resources relative to the target detection task, we were also able to assess resource demands associated with automated symbolic orienting.

## Materials and Methods

### Participants

Seventeen participants were assigned to the NP group. To ensure adequate sampling with spatially predictive cues, 29 participants were assigned to the PC group.

### Apparatus and stimuli

**Figure 1:**
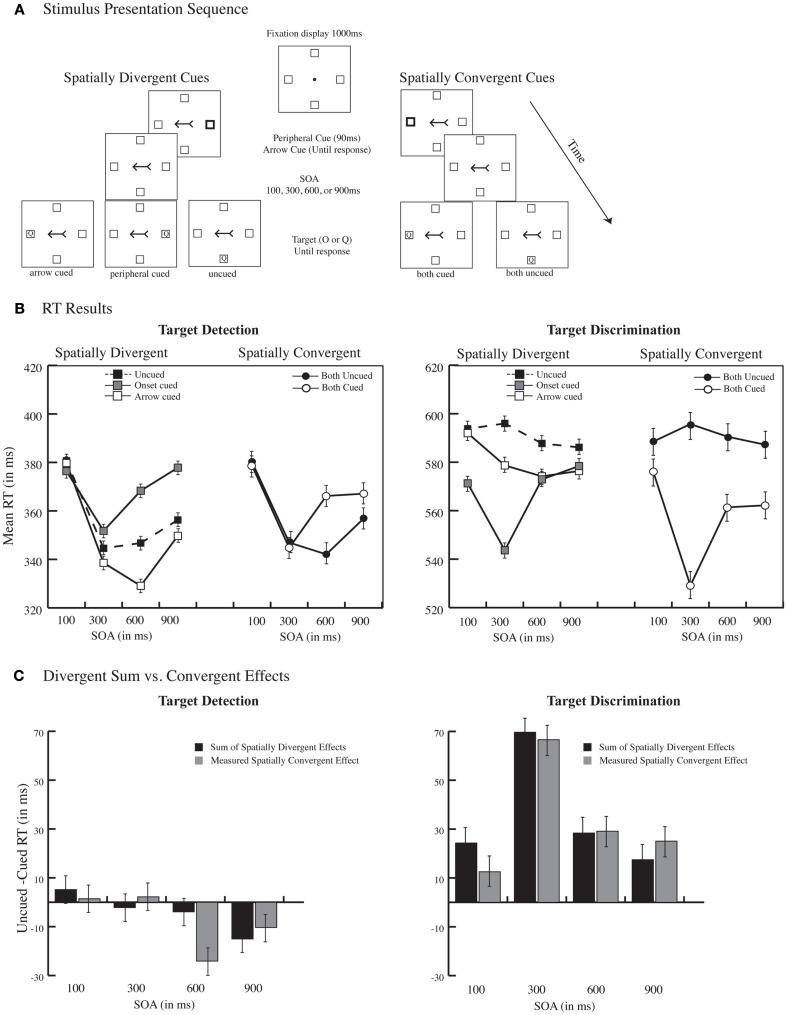
**Nonpredictive peripheral group**. **(A)** Example stimulus presentation sequence and illustration of spatially divergent and spatially convergent conditions. A 1000 ms fixation display was followed by the simultaneous presentation of two spatially nonpredictive cues, a peripheral onset, created by the thickening the outline of one of the placeholder boxes (presented for 90 ms) and a central arrow, created by attaching an arrowhead and an arrowtail to a straight line (presented until response). On any given trial, the two cues could indicate different spatial locations (spatially divergent cues) or the same spatial location (spatially convergent cues). A target letter (O or Q), demanding either a detection or a discrimination response, appeared in one of the possible four locations, and remained on the screen until a response. Note that stimuli are not drawn to scale. **(B)** RT Results. Mean correct RT is plotted as a function of cue position (divergent vs. convergent), cue validity, and SOA for target detection and target discrimination. **(C)** Divergent sum vs. Convergent Effects. The magnitude of orienting (uncued – cued RT) plotted as a function of SOA when the two cues indicated the same spatial location (convergent cues) and the sum of orienting effects when the two cues indicated different spatial location (divergent cues). Error bars depict the standard error of the difference between the means.

**Figure 2:**
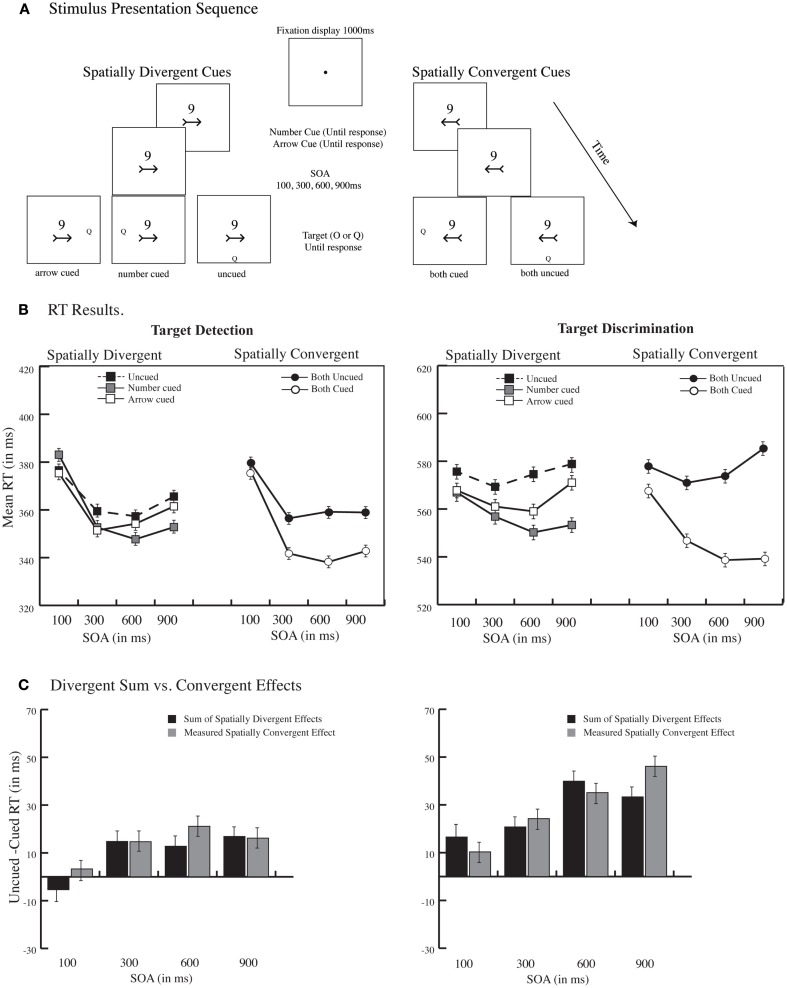
**Predictive central group. (A)** Example stimulus presentation sequence and illustration of spatially divergent and spatially convergent conditions. A 1000 ms fixation display was followed by the simultaneous presentation of two central cues, a spatially nonpredictive arrow, which indicated one of the possible four target locations equally often (*p* = 0.25), and a spatially predictive central digit (*p* = 0.77) whereby number 1 predicted a target occurring on the top, 3 a target occurring on the right, 6 a target occurring on the bottom, and 9 a target occurring on the left. On any given trial, the two cues could indicate different spatial locations (spatially divergent cues) or the same spatial location (spatially convergent cues). A target letter (O or Q), demanding either a detection or a discrimination response, appeared in one of the possible four locations, and remained on the screen until a response. Note that stimuli are not drawn to scale. **(B)** RT Results. Mean correct RT is plotted as a function of cue spatial position (divergent vs. convergent), cue validity, and SOA for target detection and target discrimination. **(C)** Divergent sum vs. Convergent Effects. The magnitude of orienting (uncued – cued RT) plotted as a function of SOA when the two cues indicated the same spatial location (convergent cues) and the sum of orienting effects when the two cues indicated different spatial location (divergent cues). Error bars depict the standard error of the difference between the means.

Stimuli were black line drawings presented against a white background on a 16″ monitor. Peripheral cues were created by thickening the outline of one of four 2° × 2° placeholders, positioned 7.2° away from center along horizontal and vertical planes. An arrow cue was created by combining a 2° straight line with a 1° arrowhead and arrowtail (each 1°). The number cues (9, 6, 3, and 1) and target stimuli (O and Q) subtended approximately 1.5°. Stimuli and sample sequences of events for the NP and PC groups are illustrated in Figures 1A and 2A.

### Design

In the NP group, a spatially nonpredictive peripheral onset and a spatially nonpredictive central arrow were shown. The position of the peripheral cue and the direction of the arrow were determined randomly, with the target appearing equally often at each target location (*p* = 0.25; Figure 1A). In the PC group, a spatially predictive central digit and a spatially nonpredictive central arrow were shown. The digit indicated the correct target location reliably (*p* = 0.77) while the arrow indicated the correct target location randomly (*p* = 0.25; Figure 2A). Number 1 predicted a target occurring at the top, 3 on the right, 6 on the bottom, and 9 on the left. These cue number – target relations have been shown to require endogenous attention in order for spatial orienting to occur (Ristic and Kingstone, 2006). Thus, across both conditions, the two cues could either indicate two different spatial locations, (spatially divergent cues) or the same spatial location (spatially convergent cues). As illustrated in Figures 1A and 2A, in the divergent conditions, the target could appear at one of the two cued locations (i.e., arrow cued; onset cued in the NP Condition; arrow cued; number cued in the PC condition) or at one of the two remaining uncued locations (i.e., uncued targets). In the convergent conditions, the target could appear at the location cued by both cues (i.e., cued target) or at one of the three remaining uncued locations (i.e., uncued targets). Participants completed both detection and discrimination tasks, which were counterbalanced for order.

### Procedure

Trials began with a 1000 ms fixation display. Then, the two cues appeared simultaneously indicating either different spatial locations (spatially divergent cues) or the same spatial location (spatially convergent cues). To preserve each cue’s normal cue-target timing, peripheral cues were presented for 90 ms while central cues remained on the screen for the duration of the trial. Following a random SOA of 100, 300, 600, or 900 ms, a target demanding either a detection or a discrimination response appeared at one of four possible locations. Detection responses were executed by pressing the spacebar key; discrimination responses were executed by pressing the “z” and “/” keys (target-response mapping was counterbalanced between participants). Trials terminated on response or timed out after 2600 ms. RT was measured from target onset. The intertrial interval was 700 ms. On approximately 10% of the trials, a target did not occur and participants were required to withhold a response.

Participants were instructed to maintain central fixation, to respond as fast and as accurately as possible, and were informed about, and it was confirmed that they understood, the spatial predictiveness of each cue.

Cue direction, target position, target type, and SOA were selected randomly. The detection and discrimination tasks were each composed of 1136 trials, where each task type was blocked and comprised of 16 blocks of 71 trials. Ten practice trials preceded each task.

## Results

The following counted as errors and were removed from RT analyses: Anticipations (RT < 100 ms; Detection NP = 1.88%; Discrimination NP = 0.1%; Detection PC = 1.47%; Discrimination PC = 0.008%), misses (RT > 1000 ms; Detection NP = 0.28%; Discrimination NP = 1.9%; Detection PC = 0.47%; Discrimination PC = 1.58%), incorrect key presses (Detection NP = 0.01%; Discrimination NP = 4.28%; Detection PC = 0.003%; Discrimination PC = 3.87%), and false alarms (Detection NP = 1.88%; Discrimination NP = 0.1%; Detection PC = 1.55%; Discrimination PC = 0.19%)^1^.

Our analyses were guided by two hypotheses: (1) If the two cues produce additive effects then no interference between the cues should be observed when they are divergent, while the sum of divergent effects should closely approximate the magnitude of convergent effects, and (2) If automated orienting engages attentional processes in both detection and discrimination tasks, both tasks should exhibit a similar pattern of results.

### NP (nonpredictive peripheral) group

Mean RTs for spatially divergent and spatially convergent conditions are illustrated in Figure 1B.

#### Divergent cues

Within-subjects ANOVAs with task type (detection vs. discrimination), cue validity (cued vs. uncued), and SOA compared mean correct RTs for peripheral onset and central arrow cues. All reported *t*-tests are based on paired two-tailed comparisons.

For peripheral cues, there were main effects of task type [*F*(1, 16) = 416.24, *p* < 0.0001] confirming the task difficulty manipulation, SOA [*F*(3, 48) = 19.95, *p* < 0.0001] reflecting the typical foreperiod effect, and cue validity [*F*(1, 16) = 6.54, *p* < 0.05] indicating an overall RT advantage for cued vs. uncued targets (467 ms cued RT vs. 474 ms uncued RT). Task type, cue validity, and SOA interacted [*F*(3, 48) = 8.56, *p* < 0.0001], reflecting that IOR, which emerged only at the late SOAs in the detection task, was abolished in the discrimination task, a finding that converges with many other past investigations (e.g., Danziger and Kingstone, 1999; Klein and Shore, 2000; Dukewich, 2009). There were also two-way interactions between task type and SOA [*F*(3, 48) = 4.92, *p* < 0.01] reflecting a more pronounced foreperiod effect the in detection task, and between task type and cue validity [*F*(1, 16) = 79.06, *p* < 0.0001] due to the larger validity effects for discrimination relative to detection task (24 vs. −11 ms). Two two-way *post hoc* ANOVAs conducted for each task type separately confirmed that IOR effect was significant at 300, 600, and 900 ms SOA in the detection task [100 ms SOA, *t*(16) = −1, *p* > 0.3; 300 ms SOA *t*(16) = 2.7, *p* < 0.05; 600 ms SOA *t*(16) = 4.1, *p* < 0.001; 900 ms SOA *t*(16) = 5, *p* < 0.0001] while early facilitation persisted until 600 ms in the discrimination task [100–600 ms SOA all *t*s(16) > −2.7, *p*s < 0.05] without being replaced by IOR at the latest SOA of 900 ms (*p* > 0.12).

For arrow cues, all main effects were significant (all *F*s > 44.84, *p*s < 0.0001; 465 ms cued RT vs. 474 ms uncued RT). The two-way interaction between task type and SOA [*F*(3, 48) = 17.43, *p* < 0001] reflected again a more pronounced foreperiod effect for the detection task, while the interaction between SOA and cue validity [*F*(3, 48) = 4.24, *p* < 0.1] indicated that the cuing effects increased in magnitude with SOA (100 ms SOA *t* < 1, all other *t*s > −4.9, *p*s < 0.001). No other effects approached significance (all *p*s > 0.20). In sum, across detection and discrimination tasks, the two cues produced their typical effects without interference.

#### Spatially convergent

When the cues converged spatially, all main effects were significant (all *F*s > 11.1, *p*s < 0.01). A three-way interaction between task type, cue validity, and SOA [*F*(3, 48) = 5.37, *p* < 0.01] indicated that the effects diminished with SOA in the detection task due to the emergence of IOR. Two two-way interactions between task type and cue validity [*F*(1, 16) = 46.66, *p* < 0.0001; Discrimination uncued – cued RT = 33 ms; Detection −8 ms] and between SOA and cue validity [*F*(3, 48) = 7.37, *p* < 0.001] were also reliable. *Post hoc* two-way ANOVAs conducted for each task type separately confirmed that while IOR emerged in the detection task producing an expected reversal of the cuing effect at SOAs exceeding 300 ms [SOA × cue validity interaction *F* (3,48) = 3.54, *p* < 0.05; 364 ms cued RT vs. 356 ms uncued RT] it was not reliable in the discrimination task as cued RTs were always faster than uncued RTs [cue validity main effect *F*(1, 16) = 35, *p* < 0.0001; 557 ms cued RT vs. 591 ms uncued RT].

#### Divergent sum vs. convergent cue effects

To assess whether the effects of the two cues were additive, the sum of the effects for the spatially divergent cues (computed as uncued – cued RT) was compared against the magnitude of the spatially convergent cues for each SOA. A two (detection vs. discrimination) × four (SOA) × two (divergent sum vs. convergent) ANOVA revealed that for both divergent and convergent cues, the effects were larger for the discrimination task [*F*(1, 16) = 58.84, *p* < 0.0001; 34 vs. −5 ms in the detection task] and decreased with SOA [*F*(3, 48) = 11.65, *p* < 0.0001] reflecting the IOR effect. Most critically, and as illustrated in Figure 1C, for both tasks the sum of divergent orienting effects mirrored convergent orienting effects across each SOA, resulting in no main effects or interactions (all *F*s < 1.7, *p*s > 0.2). In short, the effects of the two cues were additive with no differences across tasks.

### PC (predictive central) group

Mean RTs for spatially divergent and spatially convergent conditions are illustrated in Figure 2B.

#### Spatially divergent

For digit cues, there were main effects of task type [*F*(1, 28) = 1554.19, *p* < 0.0001], SOA [*F*(3, 84) = 20.77, *p* < 0.0001], and cue validity [*F*(1, 28) = 26.59, *p* < 0.0001; 458 ms cued RT vs. 470 ms uncued RT]; as well as an interaction between task type and SOA [*F*(3, 84) = 10.27, *p* < 0.0001] indicating a more pronounced foreperiod effect in the detection task. The task type × cue validity interaction was also reliable [*F*(1, 28) = 19.15, *p* < 0.005] reflecting larger effects in the discrimination vs. detection task (18 vs. 5.7 ms), while the SOA × cue validity interaction [*F*(3, 84) = 12.43, *p* < 0.0001] reflected an increasing validity effect with lengthening of SOA (from 1 ms at 100 ms SOA to 19 ms at 900 ms SOA), as it is typical for endogenous orienting (e.g., Muller and Rabbitt, 1989).

Similar results were obtained for arrow cues. Main effects of task type [*F*(1, 28) = 1192.5, *p* < 0.0001], SOA [*F*(3, 84) = 8.89, *p* < 0.0001], and cue validity [*F*(1, 28) = 16.89, *p* < 0.005; 463 ms cued RT vs. 470 ms uncued RT] were recorded; as were reliable interactions between task type and SOA [*F*(3, 84) = 3.76, *p* < 0.05] reflecting the steeper foreperiod effect for the detection task, and between task type and cue validity [*F*(1, 28) = 4.7, *p* < 0.05] reflecting larger cuing effects for the discrimination relative to detection task. Thus, both cues produced orienting effects that would be expected as if they were presented in isolation.

#### Spatially convergent

When the cues converged spatially, there were the usual main effects of task type, SOA, and cue validity (all *F*s > 11, *p*s < 0.0001), and interactions between task type and SOA [*F*(3, 84) = 3.8, *p* < 0.05], task type and cue validity [*F*(1, 28) = 9.825, *p* < 0.01] and between task type, SOA, and cue validity reflecting the larger increase of effects across SOAs for the discrimination task [*F*(3, 84) = 3.16, *p* < 0.05; Detection vs. Discrimination uncued – cued RT at 100 ms 3.3 ms vs. 10.3 ms; 300 ms 15 ms vs. 24 ms; 600 ms 21 ms vs. 35 ms; 900 ms 16 ms vs. 46 ms, respectively].

#### Divergent sum vs. convergent cue effects

As illustrated in Figure 2C, the sum of the divergent cuing effects again closely mirrored the rise and fall of the convergent cuing effects across SOAs and task type. A within-subjects ANOVA indicated only significant main effects of task type [*F*(1, 28) = 19.66, *p* < 0.0001] and SOA [*F*(3, 84) = 11.92, *p* < 0.0004] with no interactions (all other *F*s < 1.5, *p*s > 0.2). Thus, at each SOA, for each detection and discrimination task, the sum of divergent effects mirrored convergent cuing effects indicating additivity^2^^,^^3^.

### Between cue contrasts

Finally, we compared the effect of an arrow cue in the divergent NP condition against the effect of an arrow cue in the divergent PC condition across task type. If arrow effects are truly independent, they should not vary across NP and PC cue pairings.

The data supported this notion. A mixed effects ANOVA with cue condition (NP vs. PC) as a between-subject factor, and task type, cue validity, and SOA as within-subject factors confirmed that effects elicited by an arrow cue did not differ across NP and PC groups. The only interactions involving cue condition were those between cue condition and SOA [*F*(3, 132) = 4.97, *p* < 0.05], and between cue condition, task type, and SOA [*F*(3, 132) = 3.1, *p* < 0.05] both reflecting differences in the foreperiod effect for detection and discrimination tasks. Main effects of task type, SOA, and cue validity continued to be reliable [all *F*s > 32.87, *p*s < 0.0001] as were interactions between SOA and cue validity [*F*(3, 132) = 3.32, *p* < 0.05] and task type and cue validity [*F*(1, 44) = 4.32, *p* < 0.05] reflecting larger effects in the discrimination task.

## Discussion

For both target detection and target discrimination, the effects of a nonpredictive central arrow co-occurred with, and were independent of, both exogenous and endogenous orienting. The new detection data replicate, without exception, the findings of Ristic and Kingstone (2012) while the discrimination data show that automated symbolic orienting reflects the engagement of selective attention.

Two lines of evidence support these conclusions. First, both the NP and PC groups produced standard cuing effects even though the cues were simultaneous and even when the cues indicated different locations. Moreover, at each SOA the summation of these divergent cue effects equaled the convergent cue effects. Thus the significant effects of automated symbolic orienting, and its time course, remained the same regardless of whether an arrow was paired with an exogenous or an endogenous cue, and whether IOR was present in the detection task or absent in the discrimination task. Second, and crucially, as these effects were observed for the discrimination task as well as the detection task, the automated orienting effects are attentional in nature. Thus, nonpredictive arrows produce *attention* effects that are independent of exogenous orienting engaged by classic NP cues and endogenous orienting generated by PC cues.

Note as well that in their seminal study, Berger et al. (2005) concluded that exogenous and endogenous orienting interact when target discrimination is difficult, because they share a common underlying resource pool. Our data indicate that when target discrimination difficulty is increased in the manner used by Berger et al. cue independence persists. Thus automated orienting appears to draw on attentional resources that are distinct and independent of the attentional resources mediating exogenous and endogenous spatial attention.

Finally, and more broadly, our data suggest that the control of human attention is multifaceted and influenced by the behavioral and evolutionary significance of the incoming stimulus, previous experience, and the current goals of an individual (e.g., Corbetta et al., 2008; Ristic and Giesbrecht, 2011). Conceptualizing human attention within this expanded theoretical framework, one that recognizes its role in both perception and complex cognitive and social behavior, is an exciting prospect for future investigations.

## Conflict of Interest Statement

The authors declare that the research was conducted in the absence of any commercial or financial relationships that could be construed as a potential conflict of interest.

## Footnotes

^1^To verify that speed accuracy trade-off did not influence RT data in the discrimination task we analyzed inter participant mean percent correct for spatially divergent and spatially convergent conditions as a function of cue validity and SOA separately for NP and PC groups. Analyses returned no significant effects involving cue validity except in the spatially divergent PC condition where cued targets were discriminated more accurately than uncued targets [*F*(2, 56) = 5.15, *p* < 0.01]. Thus, a speed accuracy trade-off was excluded.

^2^To examine whether automated orienting in the discrimination task could be attributed to increased motor priming produced by arrow cues relative to peripheral onsets or symbolic digit cues, for each NP and PC condition we computed mean RTs as a function of cue direction-response location congruency. For each condition, we then analyzed mean RTs using within-subject ANOVAs as a function of cue type (arrow vs. peripheral cue in the NP case, and arrow vs. symbolic cue in the PC case), congruency (congruent vs. incongruent), SOA, and cue validity. Both analyses returned a significant main effect of congruency (both *F*s > 47, *p*s < 0.0001; NP condition 11 ms; PC condition 9 ms) and an interaction between congruency and cue validity (both *F*s > 106, *p*s < 0.0001) indicating that the congruency effect was larger for cued than uncued trials (39 ms in the NP condition and 31 ms in the PC condition). Importantly, congruency did not vary as a function of cue type in either condition (cue × congruency interactions, both *F*s < 1) cue validity (cue × congruency × cue validity interactions, both *F*s < 1) or SOA (cue × congruency × cue validity × SOA interactions, both *F*s < 1). Thus, while overall effects of cue direction – response location congruency were observed, they did not vary across the different cue types, strongly indicating that the independence of automated orienting cannot be explained by response planning effects that are unique to the arrow cue. This result is consistent with our data indicating equivalency of the cuing effects obtained using detection and discrimination responses, as motor planning does not influence responses from a simple detection task.

^3^Our data indicated that the additivity between automated, exogenous, and endogenous orienting strongly held both within and between cue pairings. We next examined whether this finding also held at a level of an individual participant. Using linear regressions for each NP and PC condition and task type (detection and discrimination) we entered the individual sum of divergent cue effects, averaged across SOAs as a predictor of the individual divergent effects, also averaged across SOAs. Our analyses returned a very strong indication that the additivity observed at a group level also held at an individual participant level. Specifically, the results from each of the four regressions indicated that variance observed in the convergent effects could be accounted for by the variance in the individual divergent effects (all *R*^2^ > 0.4; *F*s > 10.37, *p*s < 0.05), with a trend toward significance in the PC detection condition [*F*(1, 28) = 2, *p* < 0.1].
